# Bacterial Larvicide, *Bacillus thuringiensis israelensis* Strain AM 65-52 Water Dispersible Granule Formulation Impacts Both Dengue Vector, *Aedes aegypti* (L.) Population Density and Disease Transmission in Cambodia

**DOI:** 10.1371/journal.pntd.0004973

**Published:** 2016-09-14

**Authors:** To Setha, Ngan Chantha, Seleena Benjamin, Doung Socheat

**Affiliations:** 1 National Center for Parasitology, Entomology and Malaria Control, Ministry of Health, Phnom Penh, Cambodia; 2 Public Health, Valent BioSciences Corporation, Kuala Lumpur. Malaysia; Naval Medical Research Center, UNITED STATES

## Abstract

A multi-phased study was conducted in Cambodia from 2005–2011 to measure the impact of larviciding with the bacterial larvicide, *Bacillus thuringiensis israelensis* (Bti), a water dispersible granule (WG) formulation on the vector, *Aedes aegypti* (L.) and the epidemiology. In our studies, all in-use containers were treated at 8 g/1000 L, including smaller containers and animal feeders which were found to contribute 23% of *Ae aegypti* pupae. The treated waters were subjected to routine water exchange activities. Pupal production was suppressed by an average 91% for 8 weeks. Pupal numbers continued to remain significantly lower than the untreated commune (UTC) for 13 weeks post treatment in the peak dengue vector season (p<0.05). Suppression of pupal production was supported by very low adult numbers in the treated commune. An average 70% of the household harbored 0–5 *Ae aegypti* mosquitoes per home for 8 weeks post treatment, but in the same period of time >50% of the household in the UTC harbored ≥11 mosquitoes per home. The adult population continued to remain at significantly much lower numbers in the Bti treated commune than in the UTC for 10–12 weeks post treatment (p<0.05). In 2011, a pilot operational program was evaluated in Kandal Province, a temephos resistant site. It was concluded that 2 cycles of Bti treatment in the 6 months monsoon season with complete coverage of the target districts achieved an overall dengue case reduction of 48% in the 6 treated districts compared to the previous year, 2010. Five untreated districts in the same province had an overwhelming increase of 352% of dengue cases during the same period of time. The larvicide efficacy, treatment of all in-use containers at the start of the monsoon season, together with treatment coverage of entire districts interrupted disease transmission in the temephos resistant province.

## Author Summary

*Aedes aegypti* is responsible for global dengue outbreaks in the recent years. It is also the vector of Zika virus, which may be associated with the thousands of microcephaly cases in Latin America. At the moment, dengue prevention and control solely depends on vector control measures. *Ae aegypti* thrives in all types of artificial water storage containers. Countries lacking piped water systems and depending on water storage containers are required to implement preventive measures regularly. Temephos has been the mainstay of *Ae aegypti* larval control for 40 years in the Asian region. Temephos resistance is established in the vector population. Here in Cambodia, we assessed a bacterial larvicide, *Bacillus thuringiensis israelensis* (Bti) strain AM65-52 in communes and districts with temephos resistant mosquito population. A single dose of Bti treatment was sufficient to suppress the pupal and adult vector population for three months. Moreover, two cycles of Bti treatment over six months suppressed disease transmission in the treated districts. As this study was conducted over multiple years in multiple sites, we know that these data are reproducible. Our data confirm that Bti strain AM65-52 is an efficient ammunition, and when used with complete coverage of the target habitats it can prevent dengue outbreaks.

## Introduction

Cambodia located in Southeast Asia has a tropical monsoon climate with distinct dry (November–April) and wet (May–September) seasons. Dengue fever (DF) is widely prevalent in Cambodia with year-round transmission, but it peaks during the rainy season which is accompanied with high humidity. Since the 1990’s, there have been nationwide cyclical dengue outbreaks, once every 3 to 4 years [1]. Transmission is caused by all 4 serotypes of dengue virus [2]. The principal dengue vector is *Aedes aegypti* (L) [3]. The vector population is supported throughout the year by the presence of various types of domestic water containers that are used for daily household water consumption.

In 1995 the National Dengue Control Program (NDCP) was established and has since used temephos as the primary means of vector control, targeting areas with high population density or dengue high-risk areas [1]. Temephos is applied twice per year during the wet season. An estimate of 66,000 kg of temephos is used to cover 1.7 million containers per treatment in 22 provinces [1].

Development of temephos resistance in field populations was first reported by Polson in 2001 [4]. In 2013, larvae collected from sentinel sites in 7 provinces exhibited different levels of mortality (11–90%) at WHO diagnostic dosage of 0.02 mg/L temephos [5]. The susceptibility status of the F2 –F4 larvae from the same provinces was further confirmed by the World Health Organization (WHO) Collaborating Centre for Vectors, Institute for Medical Research, Malaysia (IMR) with 4–36% mortality at the diagnostic dosage [6].

In search of new larvicide tools, Cambodia’s National Malaria Center (CNM) evaluated VectoBac WG, a bacterial larvicide based on *Bacillus thuringiensis israelensis* strain AM65-52 (Bti), for its efficacy against *Ae aegypti*. VectoBac WG is a water dispersible granule formulation, which has been reviewed by World Health Organization Pesticide Evaluation Scheme (WHOPES) and is approved for introduction into finished drinking waters [7 – 8]. Since 2001, municipalities in Brazil used this Bti formulation as a substitute to temephos [9]. In 2004, a semi field study conducted in Phum Thmei village within Phnom Penh province showed that at the dosage of 0.4 g/50 L, effective control of *Ae aegypti* in cement containers containing river, rain and well waters was achieved for 3 months, without any water exchange activity [10].

This paper reports the results of a multi phased study that was conducted from 2005–2011. In 2005 and 2006, CNM expanded their study to an operational scale to determine the impact of Bti strain AM65-52 applications on field populations of *Ae aegypti* pupae and adults. In 2007, a large scale pilot program was carried out in Kandal Province. A post treatment survey was conducted to determine the community acceptance for this new larvicide introduced in potable waters and also the capability of the applicators to handle this new larvicide. Following these evaluations from 2005–2007, in 2010 the Ministry of Health (MOH) purchased 10 Mt VectoBac WG with the support of World Bank for the treatment of water containers in Kandal Province to control temephos resistant vector population. The larvicide was used to conduct 2 treatments per year in 2010 and in 2011. The impact of larviciding on the number of dengue cases was determined in 2011.

## Materials and Methods

### Field efficacy study in the years 2005 and 2006

Cambodia is administratively divided into 24 provinces. The provinces are subdivided into districts, which in turn are further divided into communes. Communes consist of several villages.

In 2005 (June-November) and 2006 (March-September) studies were conducted to measure the impact of VectoBac WG applications on field populations of *Ae aegypti* pupae and adults. The studies were conducted with the approval from the National Ethics Committee for Health Research of Cambodia.

### Description of the study site

The study was conducted in Peani and Ou Ruessei communes during both years. The communes were located in Kampong Tralach district of Kampong Chhnang Province at N 11° 55' 41", E 104° 42' 38". The communes were separated by a dual carriage road known as National Road 5. Peani commune measured within 5.7 km x 4.5 km (2565 ha) and had 9 villages, 1343 household and a population of 5439 people. Containers in this commune were treated with Bti. Ou Ruessei commune measuring 3.6 km x 5.6 km (2016 ha) with 9 villages, 1595 household and 7371 people was used as the untreated control (UTC).

The environment and houses in both communes were similar. They were farming communities living in rural traditional houses built on stilts. The walls were made from wood or woven dried palm leaves or rice straws. The houses were clustered in each village, with paddy fields surrounding the villages. Typically each house contained one room which was used as living/sleeping quarters in the cooler hours of the day. All other activities including cooking were conducted in the space under the house, which provides a cool place in the heat of the day. Animals were also kept in the space outside the enclosed walls of the house or below the house.

### *Ae aegypti* pupal surveys and adult mosquito collections

Surveillance was conducted once every 2 weeks, with 3 surveillance rounds in the pretreatment phase and 9 surveillance rounds in the post treatment phase. Each surveillance was conducted in 50 households per commune. The 50 houses were from 3 different villages in the commune. Each surveillance covered 50 different households. No one house was repeated in the surveillance rounds during the year, as this methodology allowed broader surveillance coverage. In 2006, the 2^nd^ year of study, a GPS unit (Garmin GPSMAP 60Cx) was used to map the houses surveyed in both communes for each of the 12 surveillance rounds.

During the 2 years of study, a total of 24 surveillance rounds were conducted in both communes using the same two entomology teams to conduct the pupal and adult surveillance. Pupal and adult populations were measured in the same household. All pupae were collected from in-use and discarded containers and were allowed to emerge in the CNM insectarium. The emerged adults were then identified.

Adult mosquitoes were collected in the field with an aspirator. A technician with a battery operated aspirator (100 cm long) swept the entire indoor for 10 minutes per house. The adults collected in the insect bag were transferred to CNM for identification and *Ae aegypti* mosquitoes were counted. In 2006, the percent (%) houses in both communes were categorized according to the numbers of collected indoor adult *Ae aegypti* mosquitoes. The categories were set at 0, 1–5, 6–10, 11–20, 21–30, 31–40, and ≥ 41 mosquitoes per household. There are no previous reports or publications to correlate the threshold adult mosquito numbers to dengue outbreaks in Cambodia or elsewhere. The authors in this study made an arbitrary decision to categorize as such to reflect the impact of Bti treatment on the infestation of indoor *Ae aegypti* adult mosquitoes.

### *Ae*. *aegypti* pupal production in different types of larval habitat

During the 2005 and 2006 surveys, the productivity of water receptacles for *Ae aegypti* pupae was determined in the untreated Ou Ruessei commune in order to characterize production by container type. The containers were categorized into 3 types, following the guidelines used by the Cambodian National Dengue Control Program (NDCP) in their annual larviciding program with temephos. The first category included key containers normally recommended by WHO for treatment, i.e. the cement jars with volume capacity 100 L to 500 L. The second category included key containers that were recommended and included in the treatment program by NDCP: cement tanks with volume capacity ≥ 200 L, earthen jars with volume capacity ≥ 80 L, metal drums (200 L volume capacity), and cooking utensils of 30–50 L. The third category were containers that were not under the annual treatment program, such as earthen jars with volume capacity ≤ 80 L, cooking utensils of ≤ 30 L volume capacity which included bamboo containers and also any other containers used for animal feed which included tires and coconut shells.

### Larviciding with *Bacillus thuringiensis israelensis* (Bti)

VectoBac WG, Bti strain AM65-52, (manufactured by Valent BioSciences, IL, USA) was used to treat the larval habitats in Peani commune in both years of the study. Lot 125–516 PG was used in 2005 and Lot 139-345-3L was used in 2006. Direct application of the product was made at 8 gm/1000 L, based on the container volume. The same team of 8 applicators worked in 4 groups, with 2 applicators per group to conduct the operation in both years. In 2005, treatment was conducted from July 25–29 and in 2006 it was conducted from May 1–5. At the start of the treatment operation, the applicators were given a review of the target larval habitats. The applicators carried about 2.5 kg material in plastic containers with lids. They dispersed the Bti material over the entire surface of each container using a pre-calibrated teaspoon. One full teaspoon (scooped flat) held 1.6 g of the WG formulation, which was sufficient to treat a 200 L container. All target larval habitats with or without water were treated, with a dose based on their volume capacity. The treated containers were marked with permanent paint as soon as the container was treated with Bti. All treated containers were subject to their routine water exchange activity.

### Years 2007, 2010 and 2011, operational dengue control program using Bti in Kandal Province

Prior to the operational programs in each year, training sessions were held for local supervisors and applicators. The training sessions were held about 2–3 days before the treatment was initiated. Leaflets describing the product, its efficacy and safety were distributed to each household. All target containers were treated and marked as described in the section above under “Larviciding with *Bacillus thuringiensis israelensis* (Bti)”.

### Year 2007, post Bti treatment survey

In 2007, the Bti treatment in Kandal Province was completed in selected communes in May. The communes were selected based on high population density and dengue incidence rate. The NDCP team from CNM conducted their routine survey for 14 days to evaluate the treatment coverage of the larviciding program. During this survey they questioned the householders on the acceptance or rejection of the new larvicide and the reason(s) for the acceptance or rejection.

### Years 2010 and 2011, evaluation of the impact of Bti treatment on dengue cases

In 2010 and 2011, the impact of larviciding with Bti on dengue transmission in Kandal Province was determined. This province has 11 districts. In each year two Bti treatments were made during the season. No other larviciding or adulticiding measures were conducted.

In 2010, the treatment covered only selected communes in the 11 districts. Communes were selected based on high population density and dengue incidence rate. While in 2011, six districts with the highest dengue incidence rate in the previous year 2010 were chosen for the treatment program. All communes in the six districts received full coverage of Bti treatment. The remaining 5 districts were not under any mosquito intervention program during this study period.

Dengue case numbers are routinely collated by NDCP in CNM from all 24 provinces in Cambodia. The health department in each province tabulates the weekly data from all government hospitals in the province and forwards the data to NDCP. NDCP also collects case numbers from 7 sentinel hospitals: Kantha Bopha Hospital and National Pediatrics Hospital from Phnom Penh city; Takeo, Kompot and Kg Cham hospitals from the 3 respective provinces; and Angkor and Javavarman VII hospitals from Siem Reap province. All reported cases are diagnosed by clinical symptoms, and some are serologically identified by Institute Pasteur, Cambodia.

The impact of larviciding with Bti on dengue transmission was not determined by using the population based epi indicator, such as Incidence Rate (IR), because the annual population census data was lacking in Cambodia. So we determined the impact of larviciding by comparing the dengue case numbers between 2 consecutive years for 3 different phases: pre Bti treatment; window; and post Bti treatment. After the Bti treatment a 4 week lapse was given to allow the existent adult mosquito population and the circulating dengue virus in human and adult mosquitoes to diminish. This 4 week period is denoted here as the ‘window’ period.

### Statistical tests

For studies conducted in the year 2005 and 2006, comparisons were made for each surveillance for mean number of containers, *Ae aegypti* pupae and adults between the untreated commune, Ou Ruessei and Bti treated commune, Peani. SPSS ver. 17 was used for this purpose. Normality tests indicated that the data was not normally distributed for both communes (p<0.05). Therefore a non-parametric test, Mann-Whitney U test was performed to determine the differences in the 3 parameters mentioned above between the Bti treated and untreated commune.

The impact of Bti treatment on dengue cases in the year 2011 was also statistically analyzed. The comparisons were made for each district for dengue cases between year 2010 and 2011 at 3 different phases: pre Bti treatment phase (weeks 1–20); window phase (weeks 21–24); and post Bti treatment phase (weeks 25–36). Test of normality indicated that the dengue cases were not normally distributed for both years (p<0.05). Therefore, the significant difference in the dengue cases between the year 2010 and 2011 was determined using a non—parametric test, the Mann-Whitney U test.

## Results

### Years 2005 and 2006 field efficacy study: *Ae aegypti* pupae and adult survey results.

The single direct application exercise to treat all in-use larval habitats in Peani commune required 19 kg of VectoBac WG in 2005 and 23 kg in 2006. More than ten thousand containers were treated in each year. Efficacy of the larviciding exercise was determined by measuring the *Ae aegypti* pupae and adult mosquito numbers. Twelve surveillance rounds were conducted in each year covering a total of 600 houses per commune. These houses comprised 38% and 45% of the total household in Ou Ruessei and Peani communes, respectively.

**Fig 1:**
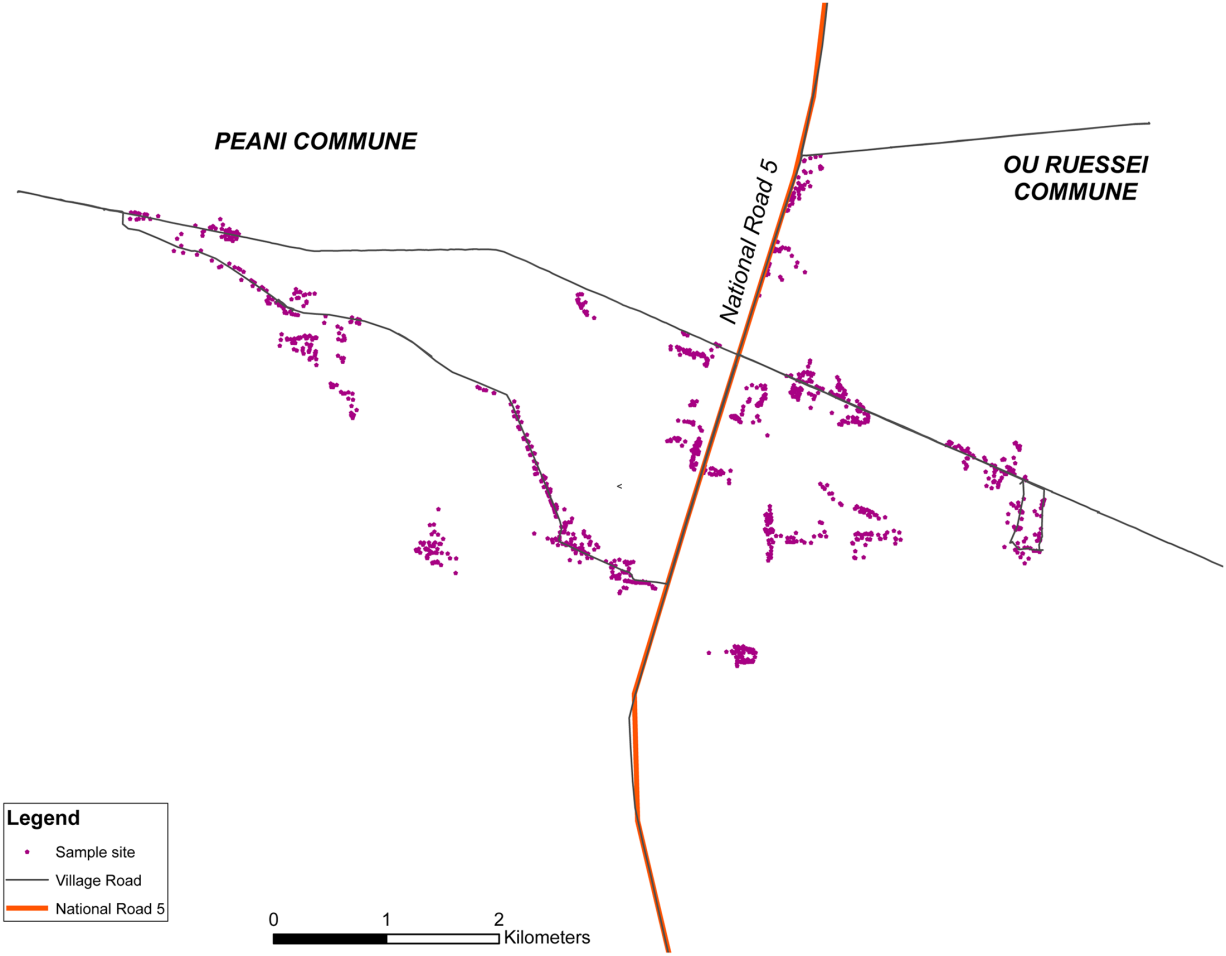
Study site in the years 2005 and 2006, Peani and Ou Ruessei communes separated by National Road 5. Each purple dot indicates a single house that was surveyed in the year 2006. A total of 12 surveillance was conducted from March-September and it covered 1200 houses from the 2 communes.

In 2006, the total 1200 houses surveyed in both communes were mapped as shown in Fig 1.

The mean number of containers surveyed in Peani and Ou Ruessei communes for each surveillance for the years 2005 and 2006 are as shown in Table 1. In 2005, it was an average of 3–4 containers per household, and in 2006 most homes had an average of 4 containers per household. In both years, for several surveillances the number of containers significantly differed between the 2 communes, and generally the difference is by one container. A significant difference of one container did not give any significant difference in the pupal numbers between the 2 communes, as observed during the pretreatment phase in 2006 (Fig 3).

**Table 1:** For Years 2005 and 2006, the Mean Number ± S.E. of Containers per Household Surveyed in Peani and Ou Ruessei Communes for *Ae aegypti* Pupae. Each surveillance covered 50 household per commune.

Surveillance	2005		2006	
Pre Treatment	Ou Ruessei Untreated Commune	Peani Bti treated Commune	Ou Ruessei Untreated Commune	Peani Bti treated Commune
**1**	2.86 ± 0.29 ^a^	2.78 ± 0.33 ^a^	4.12 ± 0.30 ^x^	3.16 ± 0.24 ^y^
**2**	2.56 ± 0.17 ^a^	2.76 ± 0.25 ^a^	4.3 ± 0.29 ^x^	3.82 ± 0.30 ^x^
**3**	2.52 ± 0.19 ^a^	2.98 ± 0.32 ^a^	4.14 ± 0.35 ^x^	5.22 ±0.42 ^y^
**Post Treatment**				
**1**	2.74 ± 0.24 ^a^	4.8 ± 0.5 ^b^	3.56 ± 0.26 ^x^	4.26 ± 0.31 ^x^
**2**	3.7 ± 0.31 ^a^	4.4 ± 0.46 ^a^	4.4 ±0.30 ^x^	3.96 ± 0.37 ^x^
**3**	4.22 ± 0.34 ^a^	3.16 ± 0.20 ^b^	4.14 ± 0.33 ^x^	4.06 ± 0.30 ^x^
**4**	3.72 ± 0.29 ^a^	3.80 ± 0.25 ^a^	5.08 ± 0.42 ^x^	3.4 ± 0.22 ^y^
**5**	4.16 ±0.31 ^a^	2.98 ± 0.35 ^b^	4.08 ± 0.33 ^x^	3.78 ± 0.31 ^x^
**6**	3.76 ± 0.36 ^a^	2.9 ± 0.25 ^a^	6.24 ± 0.41 ^x^	5.24 ± 0.46 ^y^
**7**	4.68 ± 0.35 ^a^	4.08 ± 0.39 ^a^	4.88 ± 0.35 ^x^	3.48 ± 0.22 ^y^
**8**	4.30 ± 0.41 ^a^	3.10 ± 0.30 ^b^	4.24 ± 0.30 ^x^	3.84 ± 0.32 ^x^
**9**	3.68 ± 0.29 ^a^	2.48 ± 0.19 ^b^	4.68 ± 0.37 ^x^	3.88 ± 0.33 ^y^

^**a,b**^ **or** ^**x,y**^: Indicates significant difference of the number of containers between the 2 communes for the corresponding surveillance (p <0.05).

**Fig 2:**
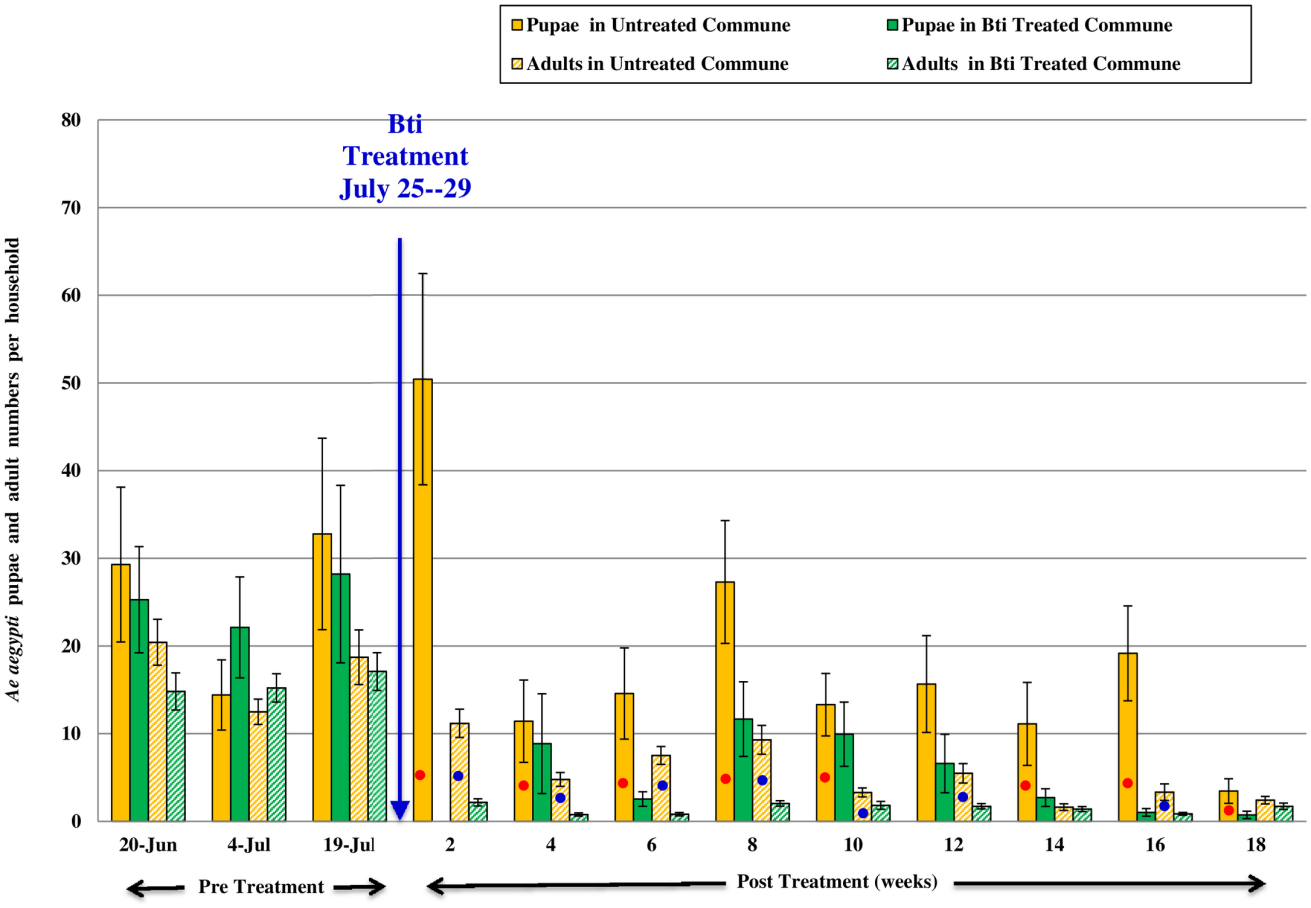
For the year 2005, mean numbers ± S.E. (per household) of *Ae*. *aegypti* pupae and adults from Ou Ruessei, the untreated commune, and Peani, Bti strain AM65-52 treated commune. The red dot indicates that the *Ae*. *aegypti* pupae significantly differed between the untreated and Bti commune for the respective surveillance (p<0.05). The blue dot indicates that the *Ae*. *aegypti* adults significantly differed between the untreated and Bti commune for the respective surveillance (p<0.05).

**Fig 3:**
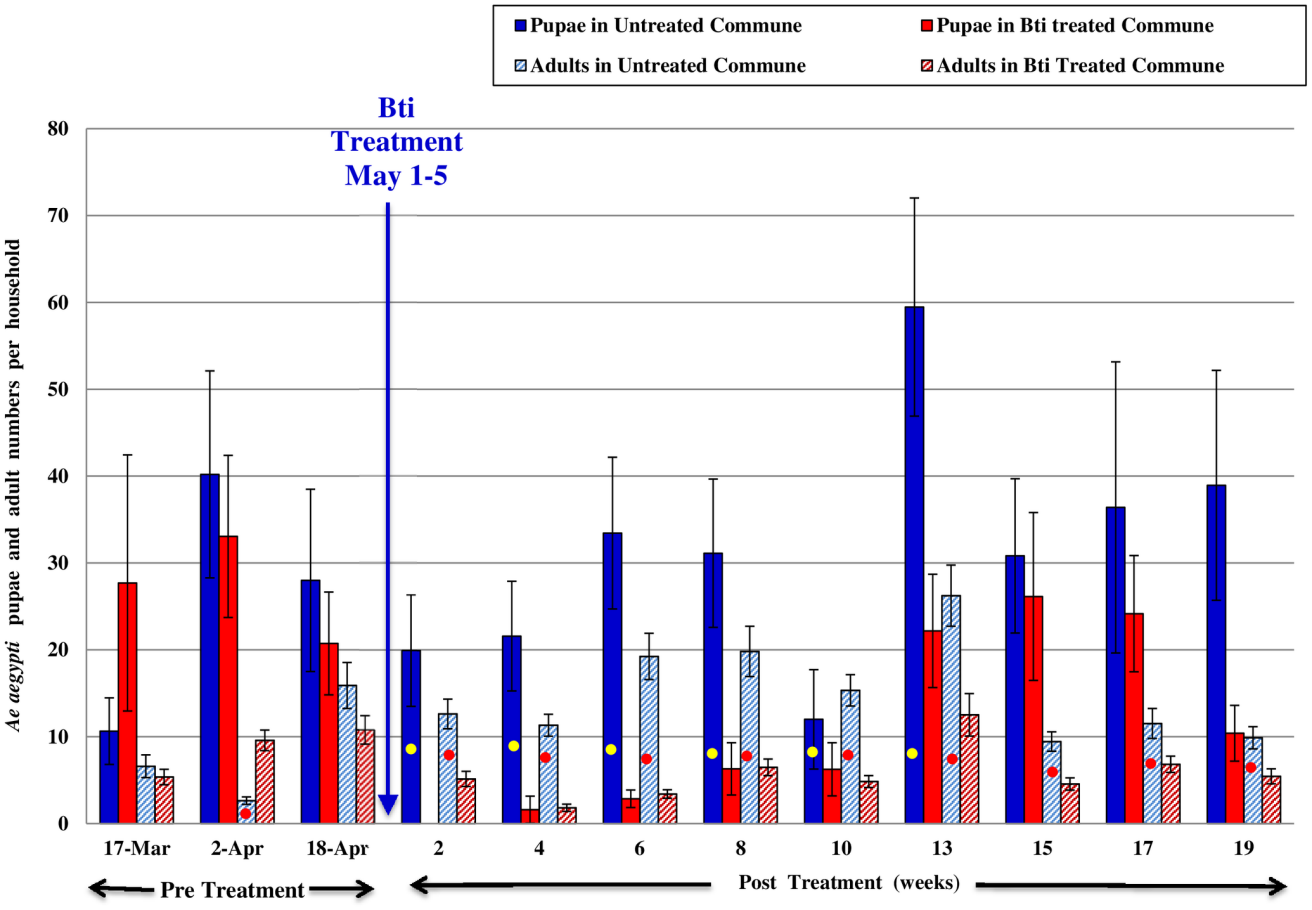
For the year 2006, mean numbers ± S.E. (per household) of *Ae*. *aegypti* pupae and adults from Ou Ruessei, the untreated commune, and Peani, Bti strain AM65-52 treated commune. The yellow dot indicates that the *Ae*. *aegypti* pupae significantly differed between the untreated and Bti commune (p<0.05). The red dot indicates that the *Ae*. *aegypti* adults significantly differed between the untreated and Bti commune (p<0.05).

Mean numbers of *Ae aegypti* pupae and *Ae aegypti* adults collected per house from Peani and Ou Ruessei communes are as shown in Fig 2 for year 2005 and in Fig 3 for year 2006.

In 2005, **s**urveillance was conducted from June-November. Pre treatment surveillance was conducted from June-July and post treatment surveillance from August-November. Both communes had similar numbers of *Ae aegypti* pupae and adults during the pre treatment phase (p>0.05). Two weeks into post treatment phase no *Ae aegypti* pupae were collected from the Bti treated commune, while 50.42 ± 12.04 pupae were collected from the UTC commune. Pupae numbers remained lower in treated commune for 18 weeks post treatment in 2005.

Significant reduction of adult mosquitoes was observed in the treated commune for continuous 12 weeks following the Bti post treatment phase (p<0.05). Seasonal mosquito population reductions were observed in both the study communes beginning in August. But, the *Ae aegypti* adult numbers continued to trend lower over time in the Bti treated commune for continuous 12 weeks post treatment with 1–2 adult mosquitoes collected per house (p<0.05).

Entomological surveillance data from 2006 is presented in Fig 3. Surveillance was conducted from March-September. Pre treatment surveillance was conducted from March-April and post treatment surveillance from May-September. Both communes had similar numbers of *Ae aegypti* pupae and adults during the pre treatment phase (p > 0.05), except in the 2^nd^ pre treatment surveillance where Peani commune had significantly higher adult population at 9.58 ± 1.19 (p<0.05). Two weeks into the post treatment phase, containers in Bti treated commune had zero pupal production, and production continued to be suppressed to very low numbers for 10 weeks post treatment. After 10 weeks the numbers increased, but remained significantly lower than in untreated commune for 13 weeks post treatment, that is during the peak dengue vector season (p<0.05). The *Ae aegypti* indoor adult population was significantly suppressed from the period of Bti treatment until the end of surveillance at 19 weeks post treatment (p<0.05). The population was lowest at 1.82 ± 0.42 per house at 4 weeks post treatment. In the untreated commune the adult numbers during the peak rainfall season from 6–13 weeks post treatment (mid-June to end July) was in the average of 15–26 mosquitoes per house. In the same period there were only 3–12 mosquitoes per house in the Bti treated commune, peaking at 12.52 ± 2.45 at 13 weeks post treatment.

The effect of larviciding with Bti on suppression of indoor adult *Ae aegypti* populations was supported by data as shown in Table 2. The percent (%) houses in both communes were categorized according to the numbers of indoor adult *Ae aegypti* mosquitoes collected during the study period in 2006. During the pre treatment sampling period, Peani commune had more houses (90.67%) harboring adult mosquitoes than the Ou Ruessei commune and it also had more houses (27.34%) with higher density of adult mosquito population (≥11 mosquitoes per house) in comparison to Ou Ruessei commune. On the first month after Bti treatment, the percentage of houses with zero mosquitoes increased to 27% in the Bti treated commune and continued to remain at a far higher percentage in comparison to the untreated commune through the rainy season until September. Most of the houses (≥ 40%) in the Bti treated commune had 1–5 mosquitoes through the 4 months (May 16^th^ -September 16^th^) of post treatment. In the same months, in the untreated commune only 3–6% of the houses did not harbor any dengue vector and the remaining homes had ≥ 6 adult mosquitoes per home. During the peak rainfall season (May-July) most homes in Ou Ruessei had 11–20 *Ae aegypti* adults per home and the percent homes with ≥ 21 mosquitoes per household increased by more than 2 folds in June and July.

**Table 2:** Year 2006, Percent (%) Household (HH) in Bti Strain AM65-52 Treated Peani Commune and in Untreated Ou Ruessei Commune Categorized according to the Numbers of Indoor *Ae*. *aegypti* Adults per Household. The Bti treatment in Peani Commune was conducted from May 1–5.

YEAR 2006	Pre Treatment Phase		Post Bti Treatment Phase							
	March-April		May-June		June		July		August-September	
Number of *Ae aegypti* adults per household	Peani Commune	Ou Ruessei Commune	Peani Commune	Ou Ruessei Commune	Peani Commune	Ou Ruessei Commune	Peani Commune	Ou Ruessei Commune	Peani Commune	Ou Ruessei Commune
	% HH	% HH	% HH	% HH	% HH	% HH	% HH	% HH	% HH	% HH
**0**	9.33	17.33	27.00	4.00	19.00	4.00	12.00	3.00	17.33	6.00
**1–5**	40.00	42.67	52.00	31.00	46.00	22.00	40.00	17.00	48.67	25.33
**6–10**	23.33	16.67	14.00	14.00	25.00	17.00	22.00	23.00	16.00	36.00
**11–20**	14.00	14.67	5.00	36.00	7.00	20.00	17.00	24.00	14.00	21.30
**21–30**	10.67	3.33	1.00	9.00	3.00	15.00	5.00	9.00	4.00	6.00
**31–40**	0.67	2.00	1.00	3.00	0.00	11.00	2.00	10.00	0.00	3.33
**> 40**	2.00	3.34	0.00	3.00	0.00	11.00	2.00	14.00	0.00	2.01

The highlighted row in black indicates that most household in Peani commune had 1–5 adult *Ae aegypti* mosquitoes per household during the post treatment Bti phase.

### *Ae*. *aegypti* pupal production from different types of larval habitat

Total number of containers surveyed in Ou Ruessei commune and the total number of pupae collected which successfully emerged into *Ae aegypti* adults are shown in Table 3. A total of 2413 and 3059 containers were surveyed in 2005 and 2006, respectively. For the 2 years, 60+ (%) of the *Ae aegypti* pupae were produced by key containers of the type recommended for larviciding by WHO. The next most productive category was containers that were not recognized as key containers by WHO or NDCP and were not under the routine larviciding program. These containers produced 23% of the pupae, with an average of 3732 pupae per study period.

**Table 3:** Years 2005 and 2006, *Aedes aegypti* Pupae Production from Different Types of Larval Habitats in Ou Ruessei Commune.

Water receptacles categorized for the annual larviciding program	Year 2005		Year 2006	
	Number of receptacles	Number of *Ae aegypti* Pupae	Number of receptacles	Number of *Ae aegypti* Pupae
		(% container productivity)		(% container productivity)
**Key containers as recommended by WHO**	1048	8944	1329	11146
		(63.74)		(60.17)
**Key containers as recommended by NDCP**	196	1818	282	3186
		(12.96)		(17.20)
**Containers not under the treatment program**	1169	3271	1448	4193
		(23.31)		(22.63)
**TOTAL**	**2413**	**14033**	**3059**	**18525**

### Year 2007, post Bti treatment survey in Kandal Province

The Bti treatment was done over 14 days in 58 communes in 11 districts from May 5–19. A 2.9 metric tons Bti material was applied in 68,241 households with 461,693 containers. A post treatment survey was conducted by the NDCP team from CNM in 1299 households and they observed through 7864 containers. A 95.55 ± 1.38% of the households accepted the Bti treatment because the larvae died within 2 hours and the treated waters did not undergo any physical change in color and odor. A 92.45 ± 1.16% of the containers were treated, showing the ability of the applicators to recognize and treat the many and varied larval habitats.

### Years 2010 and 2011, evaluation of the impact of Bti treatment on dengue cases

In 2010, two Bti treatment cycles were conducted, in June and in September. Both treatments took 14 days each and covered only selected communes in the 11 districts in Kandal province. The first treatment covered 47 communes in 11 districts with 2.09 metric tons of Bti material to treat 532,745 containers in 96,528 households. The second treatment covered 20 communes in 4 districts with 0.75 metric tons to treat 303,017 containers in 24,220 households.

We were not able to determine the impact of Bti treatment on dengue transmission in 2010 because several of the recorded dengue cases had incomplete patient address i.e. the records stated the district name, but not the name of the commune/village (within the district) where the patient resides. This prevented us from knowing the origin of the transmission, whether from a Bti treated or untreated commune.

In 2011, six districts with the highest dengue incidence rate in 2010 were larvicided with Bti. The entire six districts with 59 communes were treated with the first cycle in May, followed by a second cycle in August. The first cycle used 2.63 metric tons of Bti material for 754,098 containers in 85,239 household. The second cycle used 2.77 metric tons to treat 754,111 containers in 85,257 households. So in 2011, the pre Bti treatment phase was from weeks 1–20. The next 4 weeks was the window period, weeks 21–24. The impact of larviciding in 2011 was measured by comparing dengue case numbers between 2010 and 2011 for weeks 25–36, during the peak dengue season.

**Fig 4:**
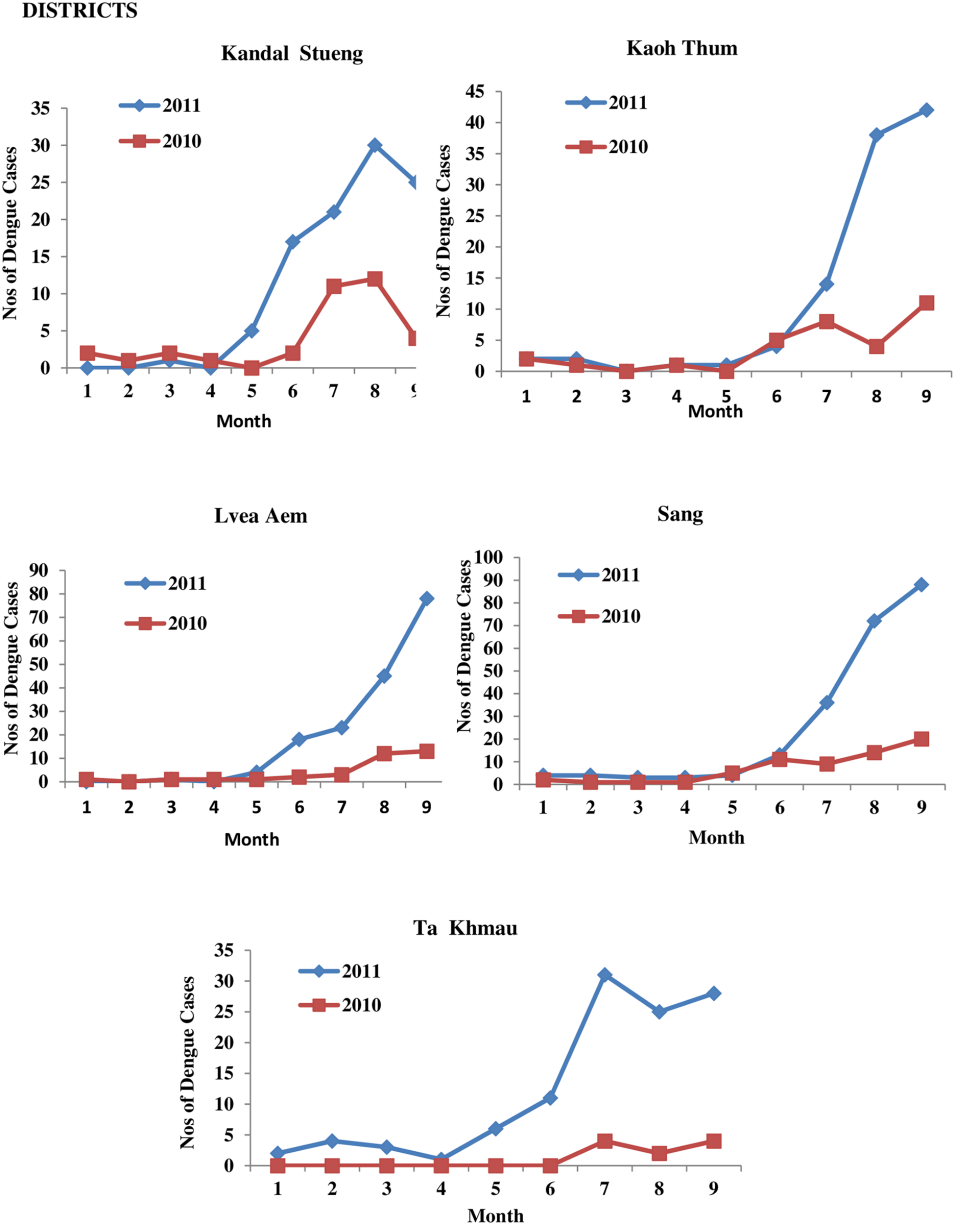
Number of dengue cases from January-September for the years 2010 and 2011 in each of the 5 districts which were not treated with Bti in 2011.

**Fig 5:**
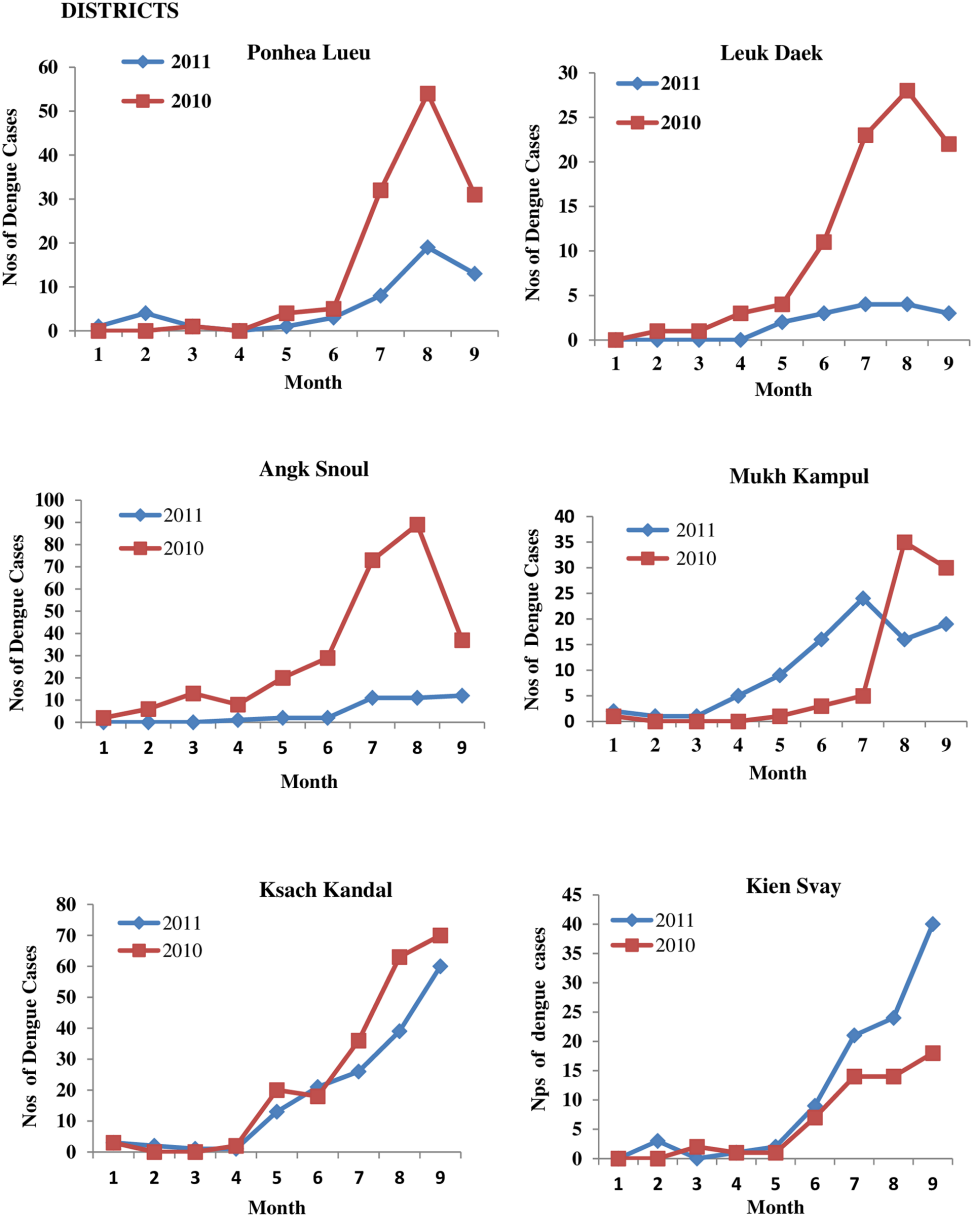
Number if dengue cases from January-September for the years 2010 and 2011 in each of the 6 districts treated with Bti in 2011.

In 2011, the 5 untreated districts had an increase in dengue cases (Fig 4), with a significant peak of 351.52% for weeks 25–36 (p < 0.05) (Table 4). In 2010 from weeks 1–36, these same 5 districts had lower number of dengue cases than the 6 districts which were used for Bti treatment in 2011 (Table 4). As for the 6 Bti treated districts during the weeks 25–36, a significant overall reduction of 47.48% cases was achieved (p<0.05) (Fig 5 and Table 4). Three of the Bti treated districts (Ponhea Lueu, Leuk Daek, Angk Snoul) had a 66–85% reduction. But, one of the district, Kien Svay had an 83% increase and this was due to untreated communes in the district. These communes were not treated as they administratively belonged to an untreated district.

**Table 4:** Summary of Dengue Cases from 11 Districts in Kandal Province for the Years 2010 and 2011.

Kandal Province	Epi Weeks	Number of dengue cases	
		Year 2010	Year 2011
**From 5 districts which did not receive any larvicide or adulticide treatment in the year 2011.**	1–20	24 ^a^	51 ^b^
	21–24	20 ^a^	63 ^b^
	25–36	132 ^a^	596 ^b^
**From 6 districts which were treated in the year 2011 with Bti strain AM65-52.**	1–20	94^a^	56 ^a^
	(Pre Bti treatment phase)		
	21–24	73 ^a^	54 ^a^
	(Window period)		
	25–36	674 ^a^	354 ^b^
	(Post Bti treatment phase)		

^**a,b**^: Indicates significant difference of the number of dengue cases between year 2010 and year 2011 for the corresponding epi weeks (p <0.05).

## Discussion

Dengue is endemic in the tropics and subtropics, with as many as 400 million infections globally per year. The dengue virus is transmitted by *Ae aegypti*, *Ae albopictus* or any other related *Aedes* species. For the moment there is no specific treatment for the disease, nor any suitable vaccine to prevent infection by any one of the four dengue viruses. Thus, the protective initiative to prevent dengue or subdue the transmission relies completely on mosquito vector control. To date, there is only one published paper describing reduction in vector populations with an impact on dengue transmission following larviciding with Bti [11]. The study was in a dengue endemic site in Malaysia and it achieved dengue suppression by larviciding with VectoBac WG (Bti strain AM65-52) [12]. Bti was applied by spray application into all target larval habitats. The adult vector population was suppressed to below 10% ovitrap index (OI) and no transmission was reported during the peak dengue season. It was contrary in the untreated site, with more than 40% OI and 15 serologically confirmed dengue cases.

This paper reports a multi phased study conducted in Cambodia from 2005–2011 and it would be the second report confirming that larviciding with Bti strain AM65-52 suppressed the adult dengue vector population in the peak season in the treated areas and also impacted the dengue transmission in the treated districts.

Cambodian economy is heavily dependent on agriculture, keeping the masses in suburban and rural areas. Water supply to these areas remains a challenge. Homeowners are dependent on waters collected from rain, groundwater and river. The waters are commonly stored in >200 L cement containers. Smaller in volume earthen containers are also found in the household vicinity together with animal feeders. A preceding semi-field study conducted in a village in Phnom Penh, Bti strain AM65-52 at 8 g/1000 L was found to significantly suppress the formation of *Ae aegypti* pupae in all 3 water types for 10–12 weeks (p<0.05) [10]. This same dosage was used for the 2 years study in 2005 and 2006. The Bti treated waters in 10,000+ containers per year was exposed to frequent flooding from rainfall, UV light, ambient temperature, and routine domestic activities. The long residual efficacy at 8 g/1000 L could be due to the nature of the formulation, enhanced by the container material. Almost all containers in the test commune were made from cement and clay. As soon as the applicator dispersed the Bti evenly across the water surface, the granules sediment to the base of the containers and some could have been embedded into the porous cement and clay matrix. Significantly longer persistence of Bti toxins has been demonstrated in earthen containers than in plastic or HDPE containers, with or without regular water exchange activity [13]. When containers were emptied completely of the Bti treated water, a near complete larval mortality was still achieved on the introduction of fresh untreated water into earthen jars, and this could be due to the release of embedded Bti toxins from the container surface. The long residual efficacy could also be due to sedimentation of the dispersible granules, leaving very minimal amounts of Bti toxins in the water column to be removed during any water exchange activity. In Australia pupae suppression was achieved for 23 weeks with the same larvicide, but at a much higher dose of 400 g/1000 L in plastic containers that were exposed to rainfall [14]. In Brazil, a 11 weeks pupae suppression was achieved with 2 g/1000 L in covered containers with no water exchange activity [15]. This same dose was used by the NDCP of Brazil to treat all containers in temephos resistant areas at 60 day intervals.

Generally, in Cambodia during the monsoon season there is an increase of *Ae aegypti* larval habitats (storage containers). This is accompanied by an exponential increase in dengue cases with a peak in August and September. In our study in 2006, we did observe in the rainy season the adult *Ae aegypti* population increased by 4 to 5 folds in the untreated commune (Ou Ruessei) from mid-June to end July 2006. The seasonal abundance of the adult population could also be due to increased larval survival rate and pupation rate, as observed in a survey conducted in a neighboring country, Myanmar [16]. In 2006, the rise of the adult vector population in the peak rainfall season was well suppressed in the Bti treated commune which received the treatment in early May. The Bti treatment made at the onset of the rainy season significantly suppressed the adult mosquito population for 4 months. Most homes in the treated commune had 0–5 mosquitoes per household. On the contrary most homes in the untreated commune had more than ≥ 6 mosquitoes per household. It is necessary that the larvicide distribution is conducted at the onset of the rainy season to curb the rise of the adult vector population during the rainy season, thus preventing outbreaks. In Cambodia 2003, a delay in temephos distribution, intended for May-June, but conducted in July–August, accounted for outbreak of dengue cases in the village that year [17]. The success of this study was also due to treating all in-use containers, irrespective of container size and its water content. The 2 year (2005 and 2006) data confirmed that small in-use containers, e.g. jars of size ≤ 80 L and cooking utensils of ≤ 30 L including animal feed containers, contributed to 23% of *Ae*. *aegypti* pupae production. Thus, it is important that these containers to be included for larvicide treatment under the routine NDCP. Treating the smaller in-use containers did not require much effort and time, because these containers are usually found placed around the bigger containers or just behind the home among the animal dwelling. NDCP cannot expect an impact on dengue transmission if efforts and resources are invested to treat only the key containers which contribute to 80% of *Ae*. *aegypti* pupae. There will always be a population of adult mosquitoes emerging from the smaller untreated containers, which will actively bite and transmit dengue.

Dry containers were also treated with Bti, because on flooding they serve as larval habitats or the dried *Ae aegypti* eggs from the previous season in the containers will hatch into fresh larvae. Dry containers can be treated at least 8 weeks before flooding with no significant decrease in larvicide efficacy [14].

In Brazil, temephos resistance was first detected in 1999 and the resistant vector populations are widespread throughout the country. Since 2001, the resistance was managed by replacing temephos with Bti in some localities [18]. The temephos resistance ratio was reduced by 30% where Bti was used, but municipalities which continued to use temephos in spite of resistance detection had increased resistance ratios in the same period of time [19]. Temephos resistant populations are susceptible to Bti and there is no cross-resistance between temephos and Bti [20].

In Cambodia, since 2009 temephos resistance has been detected in several provinces. Beginning the year 2010, the Cambodian Ministry of Health (MOH) under the Second Health Sector Support Program (HSSP2) with financial support from World Bank purchased VectoBac WG (Bti strain AM65-52) for treatment in provinces with confirmed temephos resistance. The decision to purchase was based on the local data collected in 2005–2006 on the suppression of adult *Ae aegypti* population by Bti strain AM65-52, the high acceptance of the local community in Kandal Province for this new larvicide as shown by the post Bti treatment survey in 2007 and the success of the temephos replacement program in Brazil [9]. The first procurement was used to evaluate Bti larviciding on the interruption of dengue transmission in a pilot program in Kandal Province. The 2011 pilot operational program confirmed that 2 cycles of Bti treatment in the 6 months monsoon season with a complete coverage of the target district achieved an overall reduction of 47.5% in 6 treated districts compared to the previous year, 2010. Five untreated districts in the same province had an overwhelming increase of 351.5% during the same period of time. Subsequent purchases by the Cambodian MOH were based on the results achieved for interruption of dengue transmission in Kandal Province. In the following years the NDCP used Bti strain AM65-52 in Kandal Province, and 2 other provinces with temephos resistance, Kampong Thom and Siem Reap.

The introduction of the new larvicide in each province required intense training of the larvicide distribution team. The team consisted of hundreds of applicators and supervisors under the NDCP, who have also worked to disburse temephos. Training focused on the identification of larval habitats and the importance of treating all in-use dry and wet containers. Each team member was provided with a pocket size application guide manual. The post treatment Bti survey in 2007 showed that 93% of the larval habitats were treated, concluding that the applicators were able to work efficiently with the new larvicide.

Leaflets were also distributed to each household describing the safety and specificity of this new larvicide.

Over the years, households in Cambodia have increased and so has the number of storage water containers. Thus, Cambodia requires the annual larviciding campaign against dengue. The annual campaign is effective and cost-effective intervention to reduce epidemiologic and economic burden of dengue. The reduced number of dengue cases in 2001–2005 in comparison to 1995–2000 was attributed to intervention by larviciding with temephos. It averted dengue hospitalizations, ambulatory cases and dengue deaths, which resulted in a saving of 997 disability adjusted life years (DALYs) [21]. Widespread resistant to temephos in Cambodia requires for an alternative larvicide. This multi phased study in Cambodia from 2005–2011 concludes with good supporting evidence, that larviciding with Bti strain AM65-52 by a single dose of 8g per 1000 L in all in-use containers significantly suppresses *Ae aegypti* pupae production and adult mosquitoes for a continuous 13 weeks in the peak rainfall and vector season. During the 6 months rainfall season, two Bti treatment cycles with coverage of all larval habitats within the community will interrupt dengue transmission.
